# Simultaneous small-angle neutron scattering and Fourier transform infrared spectroscopic measurements on cocrystals of syndiotactic polystyrene with polyethylene glycol dimethyl ethers^1^

**DOI:** 10.1107/S160057671601178X

**Published:** 2016-08-04

**Authors:** Fumitoshi Kaneko, Naoki Seto, Shuma Sato, Aurel Radulescu, Maria Maddalena Schiavone, Jürgen Allgaier, Koichi Ute

**Affiliations:** a Gradute School of Science, Osaka University, Toyonaka, Osaka 560-0043, Japan; b Jülich Centre for Neutron Science, Forschungszentrum Jülich GmbH, JCNS Aussenstelle am FRM II, Lichtenbergstrasse 1, 85747 Garching, Germany; c Jülich Centre for Neutron Science JCNS(JCNS-1) and Institute for Complex Systems (ICS), Forshungszentrum Jülich GmbH, 52425 Jülich, Germany; d Department of Chemical Science and Technology, The University of Tokushima, Tokushima 770-8506, Japan

**Keywords:** cocrystals, syndiotactic polystyrene, simultaneous measurement, small-angle neutron scattering, Fourier transform IR spectroscopy

## Abstract

Syndiotactic polystyrene (sPS) is a crystalline polymer which has a unique property; it is able to form cocrystals with a wide range of chemical compounds, in which the guest molecules are confined in the vacancies of the host sPS crystalline region. Recently, it has been found that even polyethylene glycol oligomers with a molecular weight of more than several hundreds can be introduced into the sPS crystalline region. It is quite important to know how such a long-chain molecule is stored in the host sPS lattice. To tackle this issue, a new simultaneous measurement method combing small-angle neutron scattering and Fourier transform infrared spectroscopy (SANS/FTIR), which has been recently developed by the authors, was applied to an sPS cocrystal with polyethylene glycol dimethyl ether with a molecular weight of 500 (PEGDME500). The temperature-dependent changes of the SANS profile and FTIR spectrum were followed from room temperature up to 413 K for a one-dimensionally oriented SANS/PEGDME500 cocrystal sample. The intensity of the reflections due to the stacking of crystalline lamellae showed a significant temperature dependence. The two-dimensional pattern in the high *Q* region of SANS also changed depending on temperature. The combined information obtained by SANS and FTIR suggested that PEGDME500 molecules are distributed in both the crystalline and amorphous regions in the low-temperature region close to room temperature, but they are predominantly included in the amorphous region in the high-temperature region. It was also suggested by the two-dimensional SANS profile that PEGDME500 molecules in the crystalline region have an elongated structure along the thickness direction of the crystalline lamellae.

## Abstract 2

A new simultaneous measurement method combining small-angle neutron scattering and Fourier-transform infrared spectroscopy was applied to a study on a syndiotactic polystyrene cocrystal with polyethylene glycol dimethyl ether with a molecular weight of 500. It is suggested that the guest molecules in the crystalline region have an elongated structure along the thickness direction of the crystalline lamellae.

## 1. Introduction   

Syndiotactic polystyrene (sPS) is a crystalline polymer that possesses some intriguing properties. It crystallizes into several crystalline states with different conformation and lateral packing, depending on the crystallization conditions and subsequent treatments (Sorrentino & Vittoria, 2009 ▸). In addition to its polymorphism, sPS forms cocrystals, where a variety of chemical compounds are included as guests in regularly arranged vacancies formed by host sPS chains of *trans*, *trans*, *gauche*, *gauche* (TTGG) conformation (Guerra *et al.*, 2009 ▸, 2012 ▸). The sPS cocrystals can be classified into four different kinds of groups, according to the crystal system and the shape of the spaces the guest molecules occupy: monoclinic δ clathrate (Chatani *et al.*, 1993 ▸; Tarallo, Schiavone & Petraccone, 2010 ▸), monoclinic δ intercalate (Tarallo *et al.*, 2006 ▸; Petraccone *et al.*, 2005 ▸), triclinic δ clathrate (Tarallo, Petraccone *et al.*, 2010 ▸) and orthorhombic ∊ clathrate (Tarallo, Schiavone, Petraccone, Daniel *et al.*, 2010 ▸). Many different kinds of chemical compounds have already been incorporated into sPS cocrystals, such as dye (Uda *et al.*, 2005 ▸) and fluorescent (Itagaki *et al.*, 2008 ▸; Del Mauro *et al.*, 2007 ▸), photo-reactive (Stegmaier *et al.*, 2005 ▸; D’Aniello *et al.*, 2007 ▸) and paramagnetic molecules (Albunia *et al.*, 2009 ▸; Kaneko *et al.*, 2006 ▸). There is a possibility that polymer crystalline region based functional materials can be developed by combining the properties of guest molecules and the host sPS lattice (Pilla *et al.*, 2009 ▸).

It has been found that there is a strong affinity between the cavities of the sPS host lattice and the chemical compounds consisting of ethylene oxide groups, (–C_2_H_4_O–)*_n_*. The incorporation of this kind of chemical compound was first found for cyclic crown ethers (Kaneko *et al.*, 2010 ▸, 2011 ▸), and was subsequently confirmed in linear polyethylene glycols (PEGs) (Kaneko & Sasaki, 2011 ▸). Up to now, even PEGs with a molecular weight of more than 1000 have been confirmed to be able to become a guest in an sPS cocrystal (Kaneko *et al.*, 2014 ▸). It is a very interesting issue how such long-chain compounds can be introduced into the crystalline cavities in the sPS lattice and which conformation they adopt. It might be desirable to utilize the information of both overall molecular shape and local molecular structure of PEGs for approaching this issue. For this purpose, we have tried to apply a new measuring method, a simultaneous measurement technique combining small-angle neutron scattering (SANS) and Fourier-transform infrared (FTIR) spectroscopy that we developed recently (Kaneko *et al.*, 2015 ▸), to the cocrystal system of sPS with PEGs.

SANS has been employed in research that aims to analyze mesoscopic scale structures. In combination with the partial deuteration technique, SANS is able to characterize the structure of a specific part in a complex system, which means that it cannot be replaced by any other method for structural analysis. SANS has established a unique position as a powerful tool for structural investigation of soft-matter and biological systems. Furthermore, SANS has extended its presence also in the research of time-dependent structure evolution. The scattering pattern of SANS is determined by the scattering length density profile in the object under study. Therefore, it is very difficult to derive the sole solution for the arrangement of constituent components only from the scattering pattern of SANS, in particular, when the system of interest is a multicomponent system. If we could get any other structural information from the same system, it would reduce greatly the difficulty of analyzing the SANS data. For this purpose, we tried to build an experimental system that enabled us to measure the FTIR spectrum and SANS two-dimensional profile from the same sample at the same time. FTIR spectroscopy has been employed as a complementary tool to X-ray and neutron diffractometry and provides information about the concentration and conformational state of each chemical species in a measured object. Simultaneous analyses by X-ray scattering and FTIR spectroscopy have already been developed for synchrotron radiation facilities (Naylor *et al.*, 1995 ▸; Innocenzi *et al.*, 2007 ▸; Jaya Ratri & Tashiro, 2013 ▸). Similarly, the combination of FTIR spectroscopy with SANS would produce a fruitful methodology for such a simultaneous measurement system. In a previous study (Kaneko *et al.*, 2015 ▸), we tested the simultaneous experimental setup on an sPS cocrystal system and confirmed its usefulness; the information obtained from the FTIR spectrum greatly helped us to interpret the SANS profile changes.

In this study, we have investigated how the long chain of PEGs is stored in the crystalline region of an sPS cocrystal by carrying out simultaneous SANS/FTIR measurements. For this purpose, we used two strategies. One is the employment of the combination of fully deuterated host sPS and protonated guest PEG molecules, which highlights the guest PEG mol­ecule in the host polymer matrix and provides information about the distribution of PEG molecules between the crystalline and amorphous regions. The second is temperature-dependent measurement. The guest molecules in the sPS cocrystal tend to migrate into the amorphous region at elevated temperatures. Accordingly, the comparison of SANS and FTIR data between the lower- and higher-temperature regions would inform us about the structural features of PEG in the crystalline and amorphous regions. In the previous simultaneous SANS/FTIR study using a short polyethylene glycol dimethyl ether (PEGDME), triethyleneglycol dimethyl ether (TEGDME) with a molecular weight of 178, hardly any of the guest molecules remained in the amorphous region at elevated temperatures because of the high volatility (Kaneko *et al.*, 2015 ▸). In response to this point, we employed a longer PEGDME with a molecular weight of about 500 (PEGDME500). In this paper, it is shown first that the SANS profile of sPS/PEGDME500 cocrystals changes significantly depending on temperature. On the basis of the SANS and FTIR results, it is demonstrated that PEG chains take a characteristic molecular shape and orientation in the crystalline region, which is very different from that of those residing in the amorphous region.

## 2. Experimental   

### 2.1. Samples   

Fully deuterated syndiotactic polystyrene (d-sPS) (weight average molecular weight 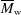 = 1.1 × 10^5^ and dispersity *Đ*_M_ = 1.9) was synthesized according to the coordination polymerization developed by Ishihara *et al.* (1986 ▸), using fully deuterated styrene with a purity higher than 98% purchased from Cambridge Isotope Laboratories. PEGDME500 was purchased from Sigma–Aldrich and used without further purification. Chloroform, acetone and their full deuterides (all purities were more than 98%) were purchased from Sigma–Aldrich and Armor Chemicals and used without further purification. Uniaxially oriented amorphous d-sPS samples, about 50 µm thick, were prepared by the following procedure: Amorphous film samples of d-sPS were obtained by quenching a melt of sPS in an ice–water bath, drawing the melt-quenched d-sPS film four times in an oil bath kept at 373 K, and clipping well oriented portions from the drawn film. The oriented amorphous films were exposed to a vapor of chloroform to give oriented samples of sPS/chloroform cocrystal. The cocrystal films were soaked in a PEGDME500/acetone mixture at about 1:1 in weight ratio for a few days to introduce the guest molecules and then kept in a vacuum oven at 313 K for an hour to remove solvent acetone, giving uniaxially oriented sPS/PEGDME500 cocrystal films.

### 2.2. Simultaneous SANS/FTIR measurement system   

**Figure 1:**
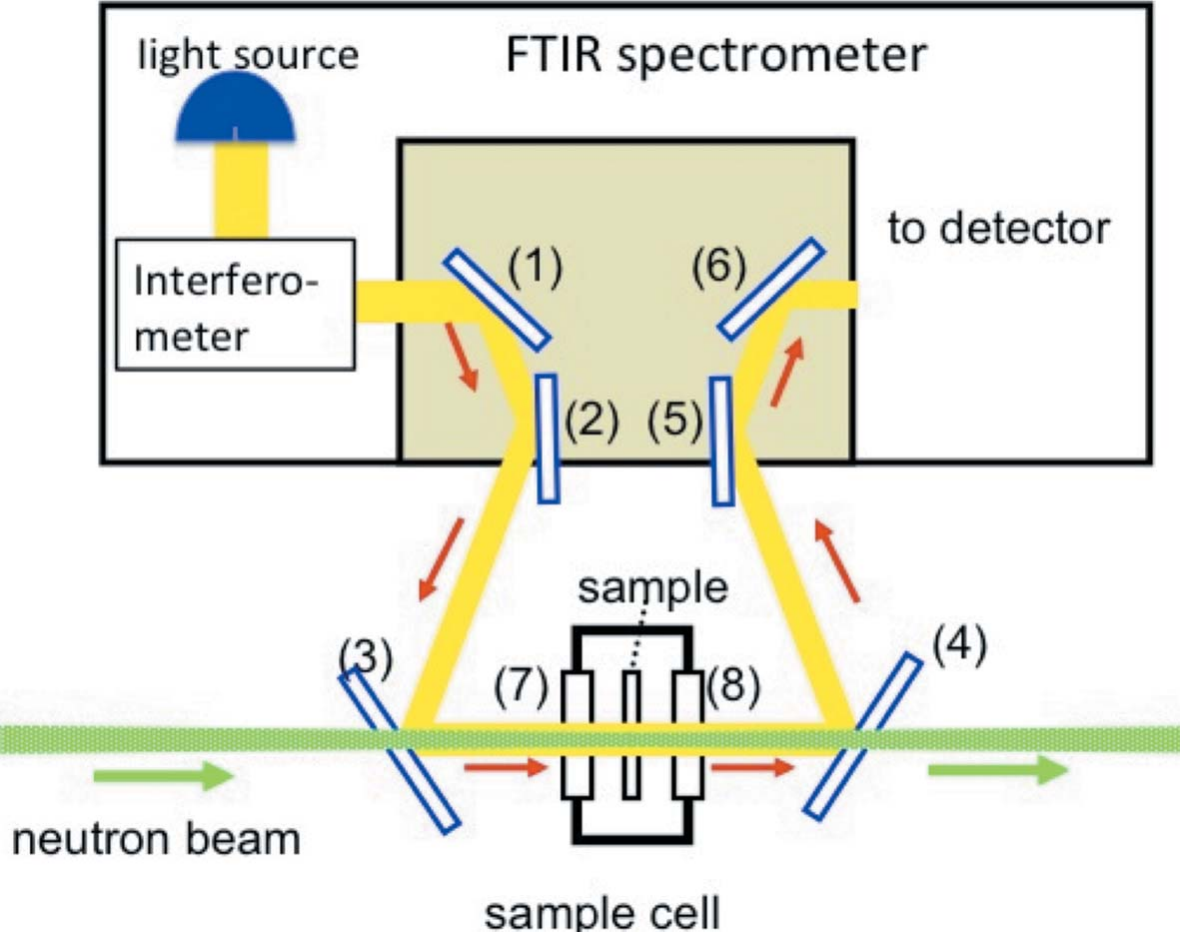
Schematic drawing of the optical system for the simultaneous measuring system combining small-angle neutron scattering with Fourier transform infrared spectroscopy. Parts 1–6 are mirrors and parts 7 and 8 are windows of the sample cell. The infrared and neutron beams are represented with yellow and green lines, respectively.

For simultaneous SANS/FTIR measurements, a device described in the previous report was used. The device, consisting of a compact portable FTIR spectrometer (Perkin­Elmer, Spectrum Two) and an optical system of our own making, was designed to be installed in the sample chamber of a SANS instrument. The concept of the simultaneous system is depicted in Fig. 1 ▸. The optical system consists of six mirrors (optical elements 1–6). Elements 3 and 4 (aluminium deposited quartz), which are irradiated by both the neutron beam (green line) and the infrared beam (yellow line), work as a beam mixer and selector, respectively. They transmit the neutron beam efficiently but act as mirrors to reflect the infrared beam. The other four elements work as mirrors only for the infrared beam. The sample cell consists of a brass body wound with a copper tube and two KBr windows (elements 7 and 8) of 2 mm thickness. Thermostated oil flows through the copper tube for temperature control. The two beams in the system are made to run on the same line by element 3 and pass through the same sample position coaxially, and then they are separated from one another with element 4, each entering into its own detector system.

#### 2.2.1. Measurements   

All the simultaneous SANS/FTIR measurements were carried out by using the KWS2 diffractometer of the Jülich Centre for Neutron Science (JCNS), outstation at Heinz Maier-Leibnitz Center (MLZ) in Garching, Germany (Radulescu *et al.*, 2012 ▸). Scattering data were obtained using a two-dimensional detector with an active area of 60 × 60 cm and 128 × 128 channels. A wavelength λ = 0.5 nm (Δλ/λ = 20%) and sample-to-detector distances of 4 and 1.35 m were chosen. The typical measured sample area was about 5 × 5 mm. The one-dimensional intensity function *I*_e_(*Q*) along the equator was obtained from the two-dimensional data corrected for detector sensitivity, instrumental noise and scattering from the empty cell, by reading pixel values and merging them with an appropriate width. The data accumulation time for each data point was 15 min. The temperature of the sample cell was controlled within an accuracy of ±0.5 K by circulating thermostated oil. The sample was kept under a slow flow of air. Transmission IR spectra were taken at a resolution of 2 cm^−1^ at 10 min intervals. The average accumulation time and the number of scans were 10 min and 128, respectively. For measuring the time dependence of IR spectra and analyzing them, commercially available software (PerkinElmer, *Timebase*) was employed. Wide-angle X-ray scattering (WAXS) measurements were carried out by using Cu *K*α radiation and an imaging plate from Fujifilm Corporation.

## 3. Results and discussion   

### 3.1. Temperature dependence of SANS profile and FTIR spectrum   

**Figure 2:**
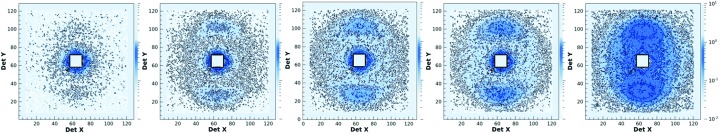
Temperature dependence of small-angle neutron scattering two-dimensional images (with a camera length of 4 m).

**Figure 3:**
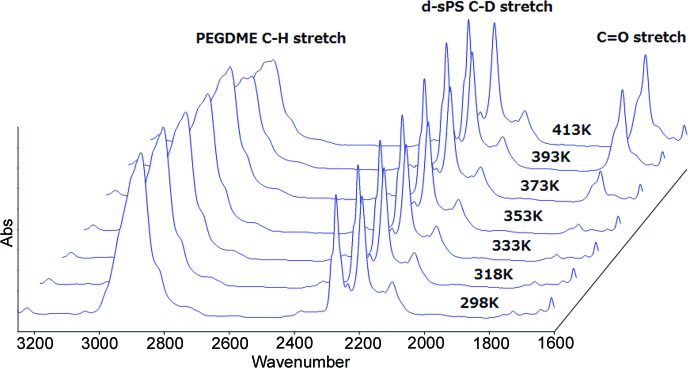
Infrared spectral changes measured in parallel with small-angle neutron scattering measurements.

The temperature dependence of the two-dimensional SANS profile is shown in Fig. 2 ▸. The two reflections due to the crystalline lamellae with a repeat period of about 100 Å appear along the meridian. The intensity of the lamellar reflection increases remarkably with temperature, in dramatic contrast to the sPS/TEGDME cocrystal whose lamellar reflections wane as the temperature increases (Kaneko *et al.*, 2015 ▸). It follows that there is an evident difference in the temperature dependence of the guest distribution between the d-sPS/TEGDME and d-sPS/PEGDME500 cocrystals, since the distribution of protonated guest molecules is the main factor to determine the scattering length density (SLD) in the host d-sPS matrix. The FTIR spectra measured in parallel with SANS two-dimensional images (Fig. 3 ▸) also suggest that the PEGDME500 component behaves in a completely different manner from the TEGDME component. For the d-sPS/TEGDME cocrystal, the bands ascribed to the C_2_H_4_O repeat unit, such as the C—H stretch around 2874 cm^−1^, decrease in intensity as the temperature increases and almost disappear around 413 K, whereas such drastic intensity changes do not take place in the d-sPS/PEGDME500 cocrystal except for a slight intensity decrease at temperatures above 373 K. With respect to the bands due to the host d-sPS, such as the C—D stretch in the region of 2300–2150 cm^−1^, the d-sPS/TEGDME and d-sPS/PEGDME500 cocrystals do not show any significant intensity changes during the course of temperature change. The difference in IR spectral changes is attributable to the volatility. The short TEGDME molecule is so volatile that it dissipates from the film as the temperature increases. On the other hand, PEGDME500 can be considered to remain in the film even at high temperatures. The slight intensity decrease of the C—H stretching band in the elevated temperature range is due to the partial degradation of PEGDME500. As shown in Fig. 3 ▸, a band around 1728 cm^−1^ appears and clearly increases in intensity with temperature. The 1728 cm^−1^ band can be assigned to the carbonyl stretching mode ν(C=O) due to the the aldehyde groups of low-mass products resulting from the thermal decomposition of polyethylene glycol, which starts even in relatively moderate conditions (Bortel *et al.*, 1979 ▸; Han *et al.*, 1997 ▸). Such low-mass products would gradually dissipate out of the film. Therefore, it can be inferred that a small amount of PEGDME500 is lost from the film owing to the thermal decomposition at elevated temperatures, which results in the slight intensity decrease of the C—H stretching band.

**Figure 4:**
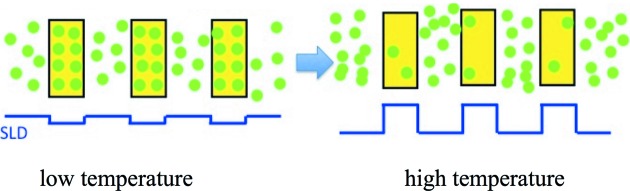
Schematic representation of the temperature-dependent change in distribution of guest molecules. The solid state of the d-syndiotactic polystyrene cocrystal containing PEGDME with a molecular weight of 500 is depicted as a one-dimensional array of crystalline lamellae (yellow boxes) and interlamellar amorphous regions. The green circles represent guest PEGDME molecules. The blue rectangle wave below represents the variation of scattering length density between the crystalline and amorphous regions.

**Figure 5:**
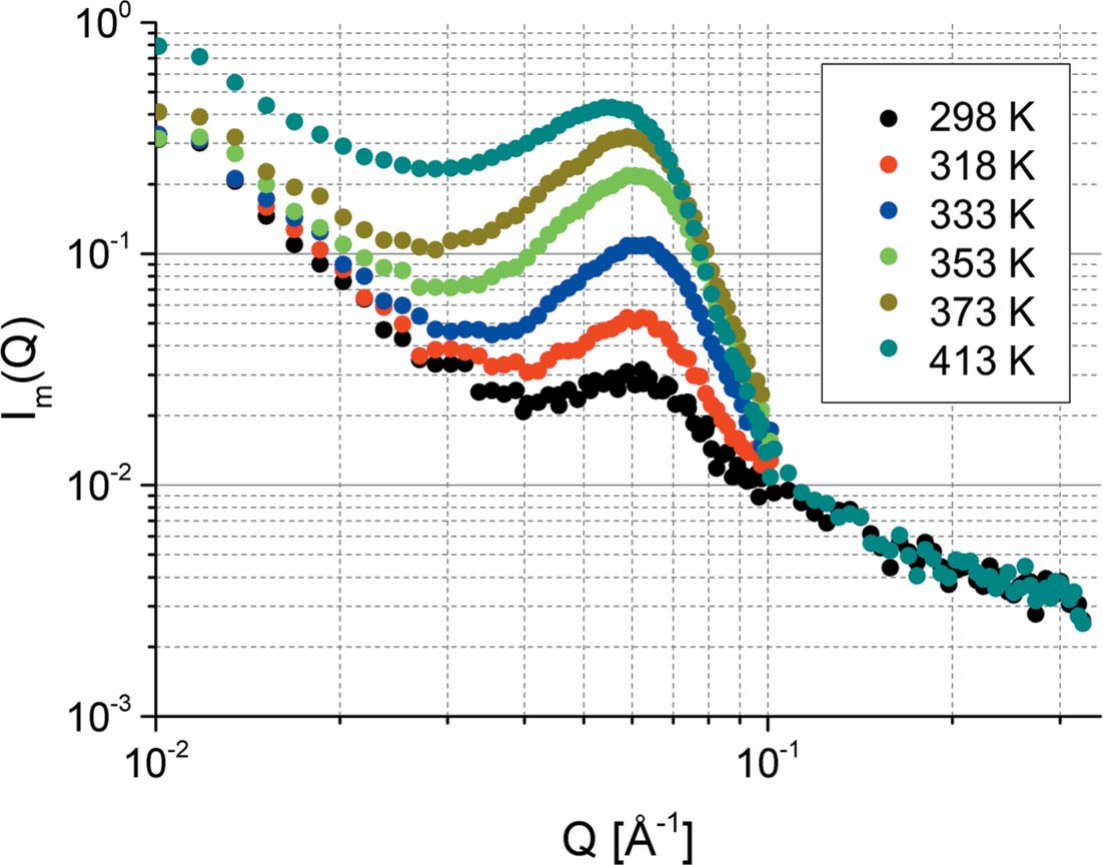
Temperature dependence of SANS one-dimensional intensity functions, *I*_m_(*Q*), along the meridian.

According to the previous studies on sPS/PEGDME cocrystals (Kaneko & Sasaki, 2011 ▸), an appreciable amount of PEGDME500 is included also in the amorphous region, which would reduce the SLD contrast between the crystalline and amorphous regions. Actually, the lamellar reflections are narrowly observable at 298 K, as shown in Fig. 2 ▸. The guest molecule in the sPS cocrystal tends to migrate into the amorphous region as the temperature increases; although stabilized enthalpically by the host lattice at lower temperatures, the guest molecule in the amorphous region gains larger entropic benefits such as higher conformational degree of freedom in the amorphous region at higher temperatures. In addition, the sPS cocrystal transforms to the γ phase at around 403 K, in which the cavities between the TTGG helices of the host sPS lattice shrink, pushing the guest molecules into the amorphous region. Taking into account these properties of sPS cocrystals and also the FTIR results, the significant intensity increase of the lamellar reflection seen in SANS can be ascribed to the migration of PEGDME500 molecules into the amorphous region; the protonated PEGDME500 mol­ecules accumulate in the amorphous region as the temperature rises, and as a result, the SLD contrast between the crystalline and amorphous regions increases, as schematically illustrated in Fig. 4 ▸. The lamellar reflection shifts towards low *Q* at the elevated temperature as shown in Fig. 5 ▸, which is ascribable to the expansion of the amorphous region caused by the guest transfer from the crystalline region to the amorphous region.

### 3.2. Anisotropic SANS profile in the high-*Q* region   

**Figure 6:**
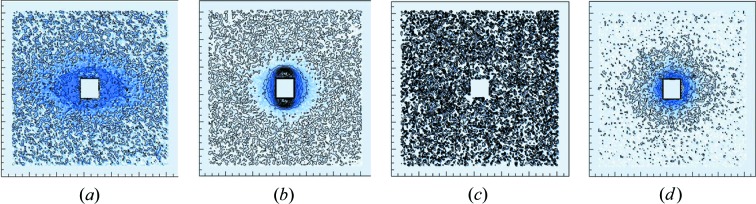
SANS two-dimensional profiles measured with a shorter camera length of 1 m (*a*)–(*c*) and 4 m (*d*). (*a*), (*b*) d-sPS/PEGDME500 at 298 and 413 K. (*c*), (*d*) d-sPS film at 298 K before guest exchange treatment.

The two-dimensional SANS profile in the high-*Q* region also shows a significant difference between low and high temperatures, as shown in Fig. 6 ▸. The d-sPS/PEGDME cocrystal exhibits a clearly anisotropic scattering profile at 398 K. The scattering intensity decreases slowly along the equator and rapidly along the meridian. The anisotropy gradually ceases as the temperature increases and the scattering profile becomes almost isotropic at 413 K; the scattering tail along the equator is not observed. As shown in Figs. 6 ▸(*c*) and 6 ▸(*d*), such anisotropic character does not appear in either the high- or the low-*Q* region before the guest exchange procedure. It follows that the SANS two-dimensional profile reflects the molecular shape of the guest PEGDME500 in the sPS film. From the temperature dependence of the two-dimensional profile, it can be inferred that PEGDME500 adopts an elongated structure along the normal of the crystalline lamellae when it resides in the crystalline region, whereas it forms an isotropic structure after migrating into the amorphous region.

### 3.3. Overall pictures of PEGDME500 in crystalline and amorphous regions   

**Figure 7:**
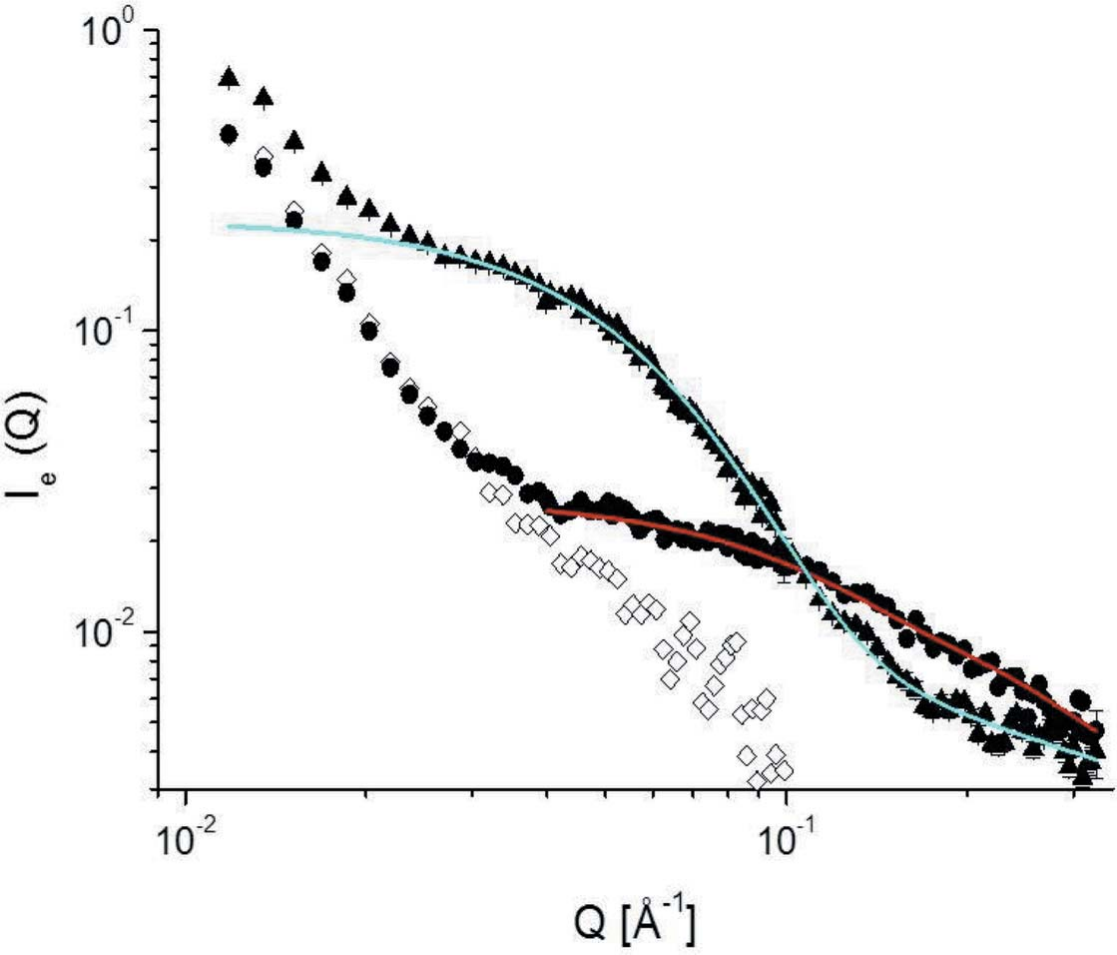
One-dimensional intensity function of d-sPS/PEGDME500 along the equator, *I*_e_(*Q*), measured at 298 and 413 K (solid circles and triangles) and of the sPS/CDCl_3_ before guest exchange (open diamonds).

**Figure 8:**
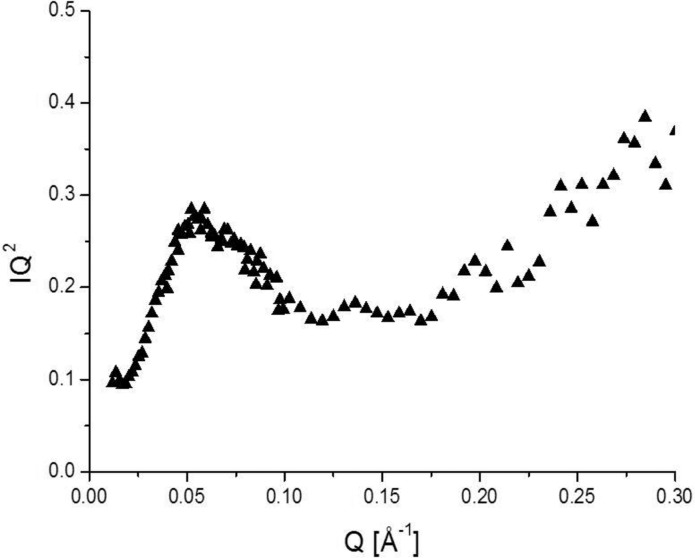
Kratky plot obtained from *I*_e_(*Q*) data at 413 K.

Contrary to the SANS profile along the meridian where the lamellar reflections emerge, there is no appreciable contribution of the lamellar stacking structure to the SANS profile along the equator. Accordingly, the molecular shape of the guest PEGDME500 is the principal factor to determine the equatorial profile. Fig. 7 ▸ shows a comparison of the one-dimensional intensity function *I*_e_(*Q*) along the equator between two measuring temperature, 298 and 413 K, together with the data for the sample before guest exchange treatment. As can be seen from this comparison, for the guest PEGDME500 there are significant differences in the *Q* dependence between 298 and 413 K. In the double logarithmic plot, *I*_e_(*Q*) decays with a slope of −1 at 298 K and −4 at 413 K, suggesting that PEGDME500 takes the form of a rod and a sphere, respectively. The fitting of the *I*_e_(*Q*) data at 298 K using a cylindrical model (Pedersen, 1997 ▸) provides a rod with a length of 40 Å and a radius of 3 Å. As for the *I*_e_(*Q*) data at 413 K, the Kratky plot (Fig. 8 ▸) is characterized by a maximum around *Q* = 0.06 Å^−1^ and a gradual increase starting around *Q* = 0.15 Å^−1^; the former suggests the existence of a compact body and the latter suggests structural looseness. Taking into account the structural features obtained from Figs. 7 ▸ and 8 ▸, we adopt a star-polymer-like structure for PEGDME500 in the amorphous region, *i.e.* an agglomerate of PEGDME500 molecules with PEG chain branches emanating from a central dense core. The radius of gyration *R*_g_ of 33 Å and the Flory exponent ν of 0.65 are derived by the fitting of the *I*_e_(*Q*) data at 413 K using the following equation given by Dozier *et al.* (1991 ▸): 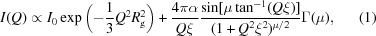
where ξ is the correlation length inside the star, μ is a parameter related to ν as μ = 1/ν − 1, Γ(μ) is the gamma function with argument μ and α is a parameter to be determined. The value of the obtained Flory exponent ν is larger than 1/2 for a random-walk chain and 3/5 for a self-avoiding random-walk chain, suggesting a rather stretched form of the PEG branches.

### 3.4. Arrangements of PEGDME500 chains in crystalline regions of the sPS cocrystal   

**Figure 9:**
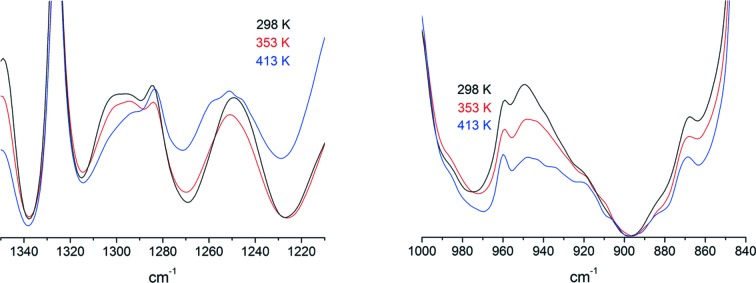
Infrared spectral changes on heating in sPS/PEGDME500, measured in parallel with SANS.

The SANS results described in the previous section strongly suggest that the PEGDME500 molecules included in the crystalline region tend to take an elongated structure in the direction perpendicular to the crystalline lamellar plane. The infrared spectrum measured in parallel with SANS shows some consistent spectral features of PEG chains. For example, the bands at 950, 1250 and 1300 cm^−1^ appear more distinctly at 298 K than at elevated temperatures, as shown in Fig. 9 ▸, which indicates that the guest PEGDME500 molecules form a more elongated structure with higher content of *trans* conformation when incorporated into the crystalline region (Deng *et al.*, 2006 ▸). PEG takes a regular helical conformation in the crystalline state, which can be regarded approximately as a uniform (7/2) helix, consisting of the regular repetition of *trans*, *trans*, *gauche* type conformation (Takahashi *et al.*, 1973 ▸). It has also been confirmed that such a regular helix conformation gradually forms when a PEG solution is cooled (Kobayashi & Kitagawa, 1997 ▸). When taking a long helical structure, PEG chains exhibit some sharp conformational regularity bands assigned to the A_2_ and E_1_ modes of the regular chain, such as the 1345 cm^−1^ A_2_ band (Yoshihara *et al.*, 1964 ▸; Matsuura & Miyazawa, 1969 ▸). The sPS/PEGDME500 cocrystal does not show such sharp conformational regularity bands. Taking these IR spectral features into account, it can be inferred that, although the PEGME500 molecules do not form a uniform regular helical structure, they take elongated molecular forms as a whole along the thickness direction in the crystalline lamellae.

The elongated molecular shape seems to be consistent with the following structural features of sPS cocrystals. First, the length of about 40 Å obtained by SANS analysis is comparable to the crystalline lamellar thickness (Kaneko, Radulescu & Ute, 2013 ▸). Second, on the guest exchange process, the new guest PEGDME500 might enter into the crystalline lamellae from the wide lamellar surface and proceed to the interior. If this happens, the new guest molecules would be apt to align perpendicular to the lamellar surface.

**Figure 10:**
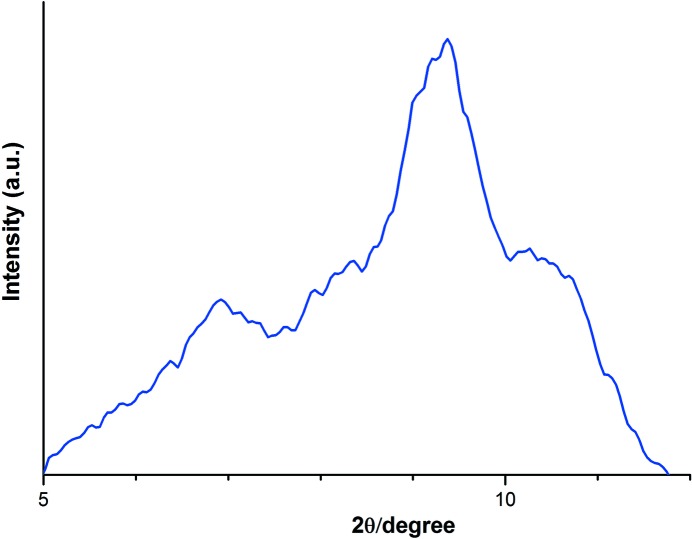
WAXS profile measured along the equatorial direction of the d-sPS cocrystal specimen after the simultaneous SANS/FTIR measurements on heating from 298 to 413 K. The reflections characteristic of the γ phase appear at 9.4 and 10.5°. The reflections at 6.9 and 8.3° are attributable to the ∊ and δ cocrystal components remaining in the specimen.

According to the previous work on sPS cocrystals with PEGDMEs (Kaneko & Sasaki, 2011 ▸; Kaneko *et al.*, 2014 ▸), the δ monoclinic clathrate having isolated cavities is generated by the guest exchange treatment on cast-grown sPS cocrystal samples. When carrying out the guest exchange procedure on a one-dimensionally stretched sPS cocrystal sample, two kinds of cocrystal structures, a major component monoclinic δ cocrystal and a minor component orthorhombic ∊ cocrystal having long tube-like hollows, are generated (Kaneko, Seto *et al.*, 2013 ▸). The coexistence of the δ and ∊ cocrystals is confirmed by the WAXS data of the d-sPS sample used for the simultaneous SANS/FTIR measurements, as shown in Fig. 10 ▸. The sample exhibits primarily the intense reflections of the γ phase, to which the δ and ∊ cocrystals have transformed during the heating process from 298 to 413 K, but also the weak reflections due to the δ and ∊ cocrystal components remaining in the sample. It is further suggested by the two-dimensional SANS profile that PEGDME500 mol­ecules in the crystalline region have an elongated structure along the thickness direction of the crystalline lamellae. The hollow tube of the orthorhombic ∊ cocrystal is suitable to accommodate the elongated PEGDME500 mol­ecules, and the contribution of the monoclinc δ cocrystal also seems to be important. As described in the previous section, the d-sPS/PEGDME500 cocrystal sample exhibits a clear anisotropic SANS profile, which suggests that the PEGDME molecule is highly oriented not only in the minor ∊-type cocrystal region but also in the major δ-type cocrystal region.

The following points would allow elongated PEGDME molecules to be incorporated into the δ cocrystal region. First, it has been confirmed that the guest molecules in the monoclinic δ cocrystal region are replaceable (Uda *et al.*, 2004 ▸; Yoshioka & Tashiro, 2003 ▸; Kaneko & Tsuchida, 2013 ▸). Chain molecules, such as *n*-alkanes, and aromatic molecules like toluene and xylene can be substituted easily, without destruction of the sPS structure. It can be inferred that the lattice of the sPS cocrystal allows such small compounds to pass through the lattice, beyond the barrier between the cavities. Second, it has been also shown that the cavities are interconnected with narrow channels and, therefore, small gas molecules (such as helium and neon) are able to pass through the cocrystal lattices (Tamai & Fukuda, 2003 ▸, 2004 ▸). Third, the potential barrier around the C—C bond connecting the phenyl side group and the main chain is low (Schaefer *et al.*, 1988 ▸), which means that the flat plane of the phenyl side group is adjustable according to the shape of the guest molecules. Fourth, the oxygen atom in the C_2_H_4_O repeat unit has a smaller diameter than the methylene CH_2_ group and the rotational potential around the C—O bond is shallower than that of the C—C bond (Anderson, 2007 ▸). With these points taken into account, it seems plausible that a flexible PEG chain occupies several cavities of the δ cocrystal at the same time, in other words, the PEGDME500 molecule would be able to straddle several cavities along the *c* axis of the sPS δ cocrystalline region.

Further, more detailed studies are necessary to obtain more detailed structure information about the packing mode of PEG chain in the sPS cocrystalline regions and to confirm the accommodation of elongated PEG molecules in both types of sPS cocrystal. We are now conducting a systematic study using a series of PEGDME molecules with a wide range of mol­ecular weight.

## 4. Conclusion   

The cocrystal structure of sPS with PEGDME500 has been studied by employing a simultaneous SANS and FTIR measuring method and the combination of deuterated polymer matrix sPS and protonated guest PEGDME500. The temperature-dependent measurements of the two-dimensional SANS profile and the FTIR spectrum provided structural information about PEGDME500. The PEGDME500 molecules stored in the crystalline region showed a clear two-dimensional anisotropic scattering profile, which indicated an elongated form perpendicular to the lamellar plane. On the other hand, the PEGDME500 molecules residing in the amorphous region exhibited an isotropic scattering profile, which suggested an agglomerate of PEGDME500 molecules having a dense core and loose arms. The FTIR spectra measured in parallel with SANS also suggested a stretched form of PEGDME500 molecules in the cocrystalline region.
